# In Silico and In Vitro Analysis of Tacca Tubers (*Tacca leontopetaloides*) from Banyak Island, Aceh Singkil Regency, Indonesia, as Antihypercholesterolemia Agents

**DOI:** 10.3390/molecules27238605

**Published:** 2022-12-06

**Authors:** Rachmawati Rachmawati, Rinaldi Idroes, Eko Suhartono, Nur Balqis Maulydia, Darusman Darusman

**Affiliations:** 1 Graduate School of Mathematics and Applied Sciences, Universitas Syiah Kuala, Banda Aceh 23111, Indonesia; 2 Department of Nutrition, Health Polytechnic of Aceh Ministry of Health, Aceh Besar 23241, Indonesia; 3 Department of Chemistry, Faculty of Mathematics and Natural Sciences, Universitas Syiah Kuala, Banda Aceh 23111, Indonesia; 4 Department of Pharmacy, Faculty of Mathematics and Natural Sciences, Universitas Syiah Kuala, Banda Aceh 23111, Indonesia; 5 Department of Medical Chemistry/Biochemistry, Faculty of Medicine, Lambung Mangkurat University, Banjarbaru 70124, Indonesia; 6 Faculty of Agriculture, Soil Science Department, Universitas Syiah Kuala, Banda Aceh 23111, Indonesia

**Keywords:** Tacca leontopetaloides, HMG Co-A reductase, in vitro, in silico

## Abstract

*Tacca leontopetaloides* (*T. leontopetaloides*) contain a number of active compounds such as flavonoids, tannins, phenolics, steroids, and alkaloids. The active compounds from plants have been shown to reduce the risk of cardiovascular disease by lowering cholesterol levels by inhibiting the enzyme 3-hydroxy-3-methylglutaryl-coenzym A (HMG-CoA) reductase activity. This study aims to investigate the potential active compounds in the ethanolic extract of Tacca tubers (*T. leontopetaloides*) from the Banyak Islands, Aceh Singkil Regency, Aceh Province both in vitro and in silico. Tacca tubers contain secondary metabolites including flavonoids, phenolics, tannins, steroids and saponins, according to phytochemical screening. In vitro investigation of ethanolic extract of Tacca tuber revealed inhibitory activity of HMG Co-A reductase with an IC_50_ value of 4.92 ppm. Based on the in silico study, active compound from the extract, namely Stigmasterol with the highest binding affinities with HMG Co-A reductase (−7.2 kcal/mol). As a comparison, the inhibition of HMG Co-A reductase activity by simvastatin with an IC_50_ 4.62 ppm and binding affinity −8.0 Kcal/mol. Our findings suggest that the ethanolic extract of Tacca tuber (T. leontopetaloides) from Banyak Islands, Aceh Province has the potential to inhibit the activity of HMG Co-A reductase.

## 1. Introduction

Cardiovascular diseases (CVDs) are the leading cause of death worldwide, with an estimated 17.9 million people dying from CVDs in 2019 [1]. According to the 2018 Basic Health Research (Riskesdas) data, incidences of heart and blood vessel disease are increasing year after year, with 15 out of 1000 persons suffering from cardiovascular disease. Hypercholesterolemia is a disorder in which total cholesterol and LDL cholesterol (low density lipoprotein) levels in the blood are increased, and incidentally, high cholesterol levels are a major risk factor for cardiovascular disease [2]. Hypercholesterolemia can raise the chance of developing atherosclerosis [3]. Statins or hydroxymethylglutaryl Co-A reductase inhibitors are routinely used to treat hypercholesterolemia. The enzyme 3-hydroxy-3-methylglutaryl-coenzym A (HMG-CoA) reductase is important in the production of cholesterol in the liver. HMG Co-A reductase catalyzes the conversion of HMG Co-A to mevalonic acid, the initial step in the production of cholesterol [4]. Inhibiting HMG Co-A reductase activity has been shown to lower cholesterol levels in both humans and animals [5]. HMG Co-A reductase inhibitors can lower intracellular cholesterol biosynthesis by inhibiting the conversion of HMG-CoA to mevalonate [6]. HMG Co-A reductase inhibitors, also known as statins, can lower total cholesterol, LDL cholesterol, and triglycerides while increasing HDL cholesterol [7]. As an effort of adding modality in treating hypercholesterolemia, researchers have explored the use of natural products. Plants produce a variety of phytochemical substances, or secondary metabolites, that are beneficial to health [8]. It was also reported that many medicinal plants have high antioxidant activity [9]. Phytochemical compounds found naturally in plants are powerful effectors of biological processes that can reduce disease risk via complimentary pathways. Moreover, in vitro studies have demonstrated that plant extract bioactive components have a hypocholesterolemic effect [5]. Flavonoids, for example, have demonstrated the ability to reduce the activity of the HMG Co-A reductase enzyme and hence prevent the synthesis of cholesterol [10]. Natural components such as medicinal herbs and nutraceuticals have the ability to limit the activity of the HMG Co-A reductase enzyme, and hence prevent cardiovascular disease and dyslipidemia. Additionally, they are easily available and cost-effective [11].

*Tacca leontopetaloides*, or Tacca, is a plant in the *Taccaceae* family that thrives in coastal locations less than 200 m above sea level (masl) [12]. The Tacca plant is thought to have originated in Southeast Asia and has since spread to tropical areas such as Africa, Asia, Australia and Oceania. It is also known as East Indian arrowroot, Polynesian arrowroot, Arrowroot, Fiji arrowroot, Tacca, Williams arrowroot and Tahiti arrowroot [13,14]. Tacca plants are generally eaten as food, but in some places, they are also used to treat various maladies [15,16]. Several earlier investigations have found glycosides, flavonoids, phenols, alkaloids, tannins, coumarins, polysaccharides, glycosides, gums, terpenes, terpenoids and Taccalins in Tacca tubers [12,16]. Ndouyang et al. [17] discovered that Tacca tubers have a good effect on fat metabolism and that administering unprocessed tubers in modest doses can lower LDL (low density lipoprotein) cholesterol levels in rats. Assatou et al. [18] discovered that Tacca tuber water extract had an antihyperlipidemic and a hypolipidemic effect in hyperlipidemic rats. However, research on the potential of Tacca tubers (*T. leontopetaloides*) in the treatment of hypercholesterolemia is still relatively limited, and no information is available on the inhibitory mechanism of HMG Co-A reductase enzyme activity by active chemicals found in Tacca tubers. The Tacca plant (*T. leontopetaloides*) grows wild in the Banyak Islands in Aceh Singkil Regency, and its use is still limited. The tuber section of the Tacca plant is often turned into starch and then used to make traditional cakes in Pulau Banyak, especially around Eid al-Fitr. There is currently no information on the active chemical content of Tacca tubers grown in the Banyak Islands of Aceh Singkil Regency. The purpose of this work is to determine the active compounds of ethanolic extract from Tacca tubers in the Banyak Islands, Aceh Singkil Regency, as well as the potential of these active compounds as HMG Co-A reductase inhibitors, both in vitro and in silico.

## 2. Results and Discussion

### 2.1. Plant Determination

**Figure 1:**
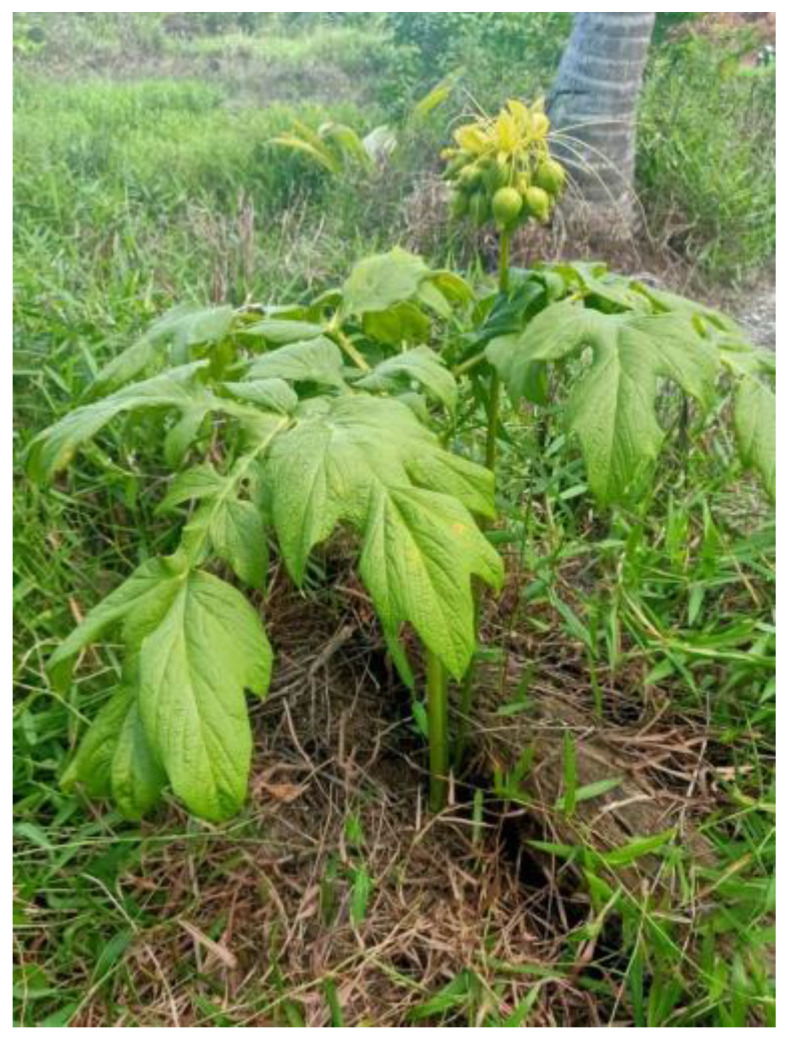
*Tacca Plant (T. leontopetaloides)*.

The results of the identification of Tacca plants (Figure 1) and their taxonomic classification are shown in Table 1.

### 2.2. Extract Yield

The extraction yield was used as an indicator of the effect of the extraction condition [19], where the yield extract indicates the amount of active compound in the extract [20]. This study revealed that the percentage yield value of ethanolic extract from Tacca tuber was 9.19%.

### 2.3. Phytochemical Analysis

Several phytochemical substances have been found to promote health by decreasing cholesterol levels and avoiding lipid oxidation, whereas others have anti-inflammatory and antiplatelet properties [21]. Low-density lipoprotein (LDL) cholesterol levels have been demonstrated to be reduced by phytochemical compounds, indicating that phytochemical compounds are effective in decreasing blood cholesterol levels and preventing the formation of undesirable cholesterol [22]. Several studies have found that active chemicals such as flavonoids, phytosterols, phenolics and alkaloids have an essential role in human health and the prevention of chronic diseases by decreasing cholesterol [23,24,25].

The identification of phytochemical substances from the extract of Tacca tuber revealed that phenolics compounds, tannins, flavonoids, steroids and saponins were detected in ethanolic extracts from Tacca tubers, unlike alkaloid compounds, which were not found in the ethanolic extract. The environmental conditions affect the synthesis and accumulation of the active compounds from the plant sample [26]. The geographical conditions, climate, genetic variation, agronomy and storage of plants are several factors that affect the active compounds of plants [27], as well as several other environmental factors, such as lighting, temperature, groundwater, soil fertility and salinity [28,29]. Some previous studies stated that Tacca tubers contain a variety of active chemicals, or secondary metabolites, including flavonoids (rutin, diosmin), saponins (chlorogenic acid, quercetin), phenols, alkaloids, tannins, coumarins, polysaccharides, glycosides, gums, terpenes, terpenoids and taccalin [30,31]. The Alkaloids, phenolics, terpenoids and tannins are phytochemical compounds that play an essential role in disease prevention, with some phytochemical substances having an effect on cholesterol metabolism, which can lower cholesterol levels [32]. Several studies have found that flavonoids can help avoid cardiovascular disease [33,34]. Tresserra-Rimbau et al. [35] found that flavonoids can reduce total cholesterol, triglycerides, low density lipoprotein cholesterol (LDL-cholesterol) and apolipoprotein B (apoB) levels while increasing HDL cholesterol and acid secretion. Catabolism of bile and lipids saponin active substances can directly block cholesterol absorption in the small intestine and indirectly impede bile acid reabsorption to lower plasma cholesterol [22]. Flavonoids, tannins, saponins, alkaloids and terpenoids have biological action as antioxidants, anti-inflammatory, anti-diarrheal, anti-ulcer and anticancer substances [36].

### 2.4. Analysis of GC-MS

Gas chromatography–mass spectrometry (GC-MS) is a technique for determining and identifying phytochemical substances in plant samples [37]. The GC-MS analysis revealed a number of active compounds, particularly fatty acids, steroids and triterpenoids in the ethanolic extract from Tacca tuber. The active compounds from the fatty acid group (i.e., hexadecanoic acid and 9,12-octadecadienoic acid) and the steroids group (i.e., campesterol, gamma sitosterol and stigmasterol) were found with varying retention times and percentages of content.

**Figure 2:**
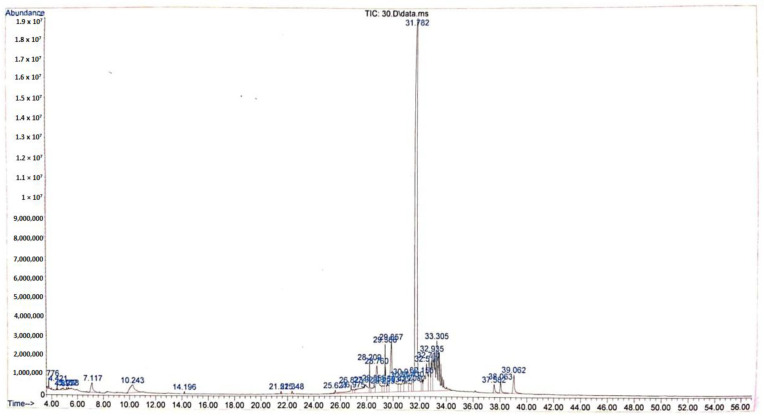
Chromatogram of Tacca tuber ethanolic extract.

The GC-MS chromatogram of ethanolic extract from Tacca tuber revealed a total of 35 peaks with different retention times (Figure 2), however there were 20 active compounds identified due to the repetition of several compounds (Table 2). The ethanolic extract from Tacca tuber contains several active compounds including 1,2-benzenedicarboxylic acid, dinonyl ester (44.39%), 9,12-octadecadienoic acid (7.0%), hexadecanoic acid (3.98%), 1-isopropyl -2-methoxycarbonyl-1-aza-cyclopropane (3.33%), phthalic acid, bis (7-methyloctyl) ester (2.74%), furancarboxaldehyde and 5-hydroxymethyl (2.12%). The ethanolic extract from Tacca tubers also contains less active compounds from the steroid group, namely campesterol (0.62%), stigmasterol (0.78%) and gamma sitosterol (1.53%).

1,2-Benzenedicaboxylic acid, butyl octyl ester is a plasticizer compound that has antimicrobial, antifouling, antioxidant and hypocholesterolemic activity [38]. The active compounds 9,12-octadecadienoic acid (linoleic acid) and hexadecanoic acid (palmitic acid) are antioxidants, anti-inflammatory, hypocholesterolemic, nemasida, hemolytic and 5-alpha reductase inhibitors [39]. Gamma-sitosterol has hypolipidemic agent [40] and antihyperglycemic [41], while campesterol has hypocholesterolemic, antidiabetic, anticancer and anti-inflammatory activity [42].

### 2.5. Molecular Docking (In Silico)

Molecular docking is a computational approach used to determine the interaction of proteins with ligands through the formation of supramolecular complexes or assemblies that can enhance or inhibit biological functions [43], inhibition of the enzyme HMG-CoA reductase by the active compound of Tacca tuber ethanolic extract and to get an idea of the mechanism that occurs between the protein target and the active compound. Molecular docking, or an in-silico test, was carried out by docking 17 active compounds of ethanolic extract from Tacca tuber (*T. leontopetaloides*) obtained by GC-MS and simvastatin as a control for the HMG-CoA reductase target protein and visualization using the Discovery Studio Visualizer 2021.

The initial stage of this research was carried out by downloading the molecular structure of the HMG-CoA reductase receptor in 3D format (.pdb) which was obtained from the Protein Data Bank, specifically the website www.rcsb.org, accessed on 3 January 2022. The molecular structure of the HMG-CoA reductase receptor used in this study is PDB ID: 2R4F [44].

The in silico study in this research demonstrates that all active compounds in the ethanolic extract from Tacca tubers have a larger free bond energy than simvastatin, indicating that all active compounds in the ethanolic extract from Tacca tubers have a lower binding affinity to the binding site of the HMG-CoA reductase enzyme when compared to simvastatin (Table 3).

**Figure 3:**
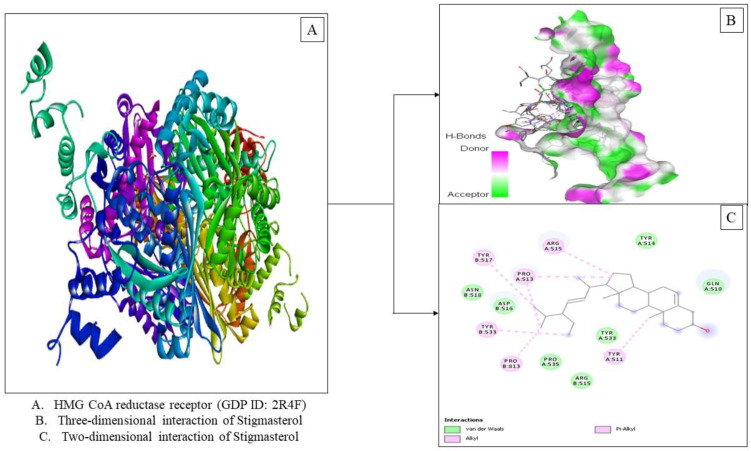
Visualization of the HMG-CoA reductase receptor docking (PDB ID: 2R4F) with stigmasterol.

The docking studies also revealed active components in the ethanolic extract from Tacca tuber with the highest binding affinity, namely stigmasterol (−7.2 kcal/mol). Interaction of stigmasterol with HMG-CoA reductase (PDB ID: 2R4F) showed the presence of van der Waals forces and hydrophobic interactions, namely alkyl and π-alkyl (Figure 3). Stigmasterol (C_29_H_48_O) is a steroid derivative characterized by the presence of a hydroxyl group in position C-3 of the steroid skeleton, as well as unsaturated bonds at positions 5–6 of the B ring and 22–23 in the alkyl substituent (Stigmasterol|C29H48O—PubChem (nih.gov, accessed on 3 January 2022) Stigmasterol is a phytosterol that has been demonstrated to reduce cholesterol absorption and the risk of cardiovascular disease [45]. According to Batta et al. [46], injection of stigmasterol can lower blood cholesterol levels and block the formation of liver cholesterol and bile acids in Wistar rats. The ethanolic extract of tacca tuber is known to contain steroid derivative compounds such as campesterol (C_28_H_48_O), Stigmasterol (C_29_H_48_O) and Gamma sitosterol (C_29_H_50_O). The presence of these compounds can help reduce of cholesterol levels. Intake of phytosterols is advised as a complementary treatment for hypercholesterolemia, and plant sterols can lower cholesterol absorption at the intestinal lumen via the Niemann-Pick C1 Like 1 (NPC1L1) transporter pathway by competitively solubilizing in mixed micelles [47].

**Figure 4:**
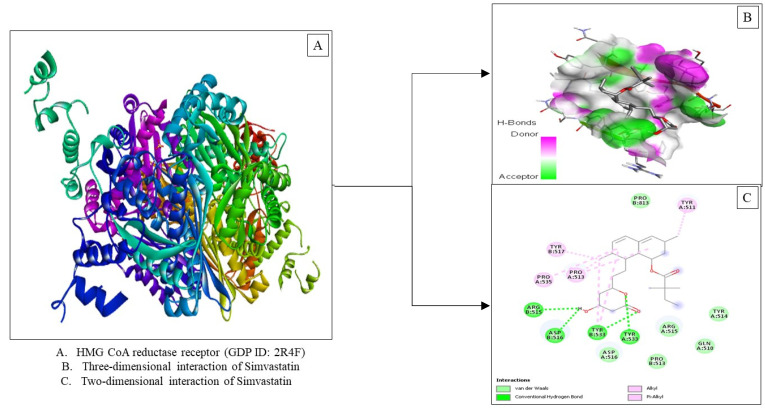
Visualization of the HMG-CoA reductase receptor docking (PDB ID: 2R4F) with simvastatin.

Based on the results of molecular docking in Table 3, Simvastatin has a binding affinity (−8.0 Kcal/mol) in the presence of three interactions, namely Van der Waals forces, hydrogen bonds and hydrophobic alkyl interactions (Figure 4). Visualization of the interaction between simvastatin and stigmasterol in the HMG-CoA reductase enzyme shows similarities in amino acid residues such as van der Waals forces, namely Aspartate: 516, Arginine: 515 and Tyrosine: 514 and hydrophobic interactions, namely Tyrosine: 511, Tyrosine: 517 and Proline: 513. The lower binding affinity value of stigmasterol could be due to the absence of hydrogen bonding as occurs in simvastatin. 

### 2.6. In Vitro Potential of HMG-CoA Inhibitor

**Figure 5:**
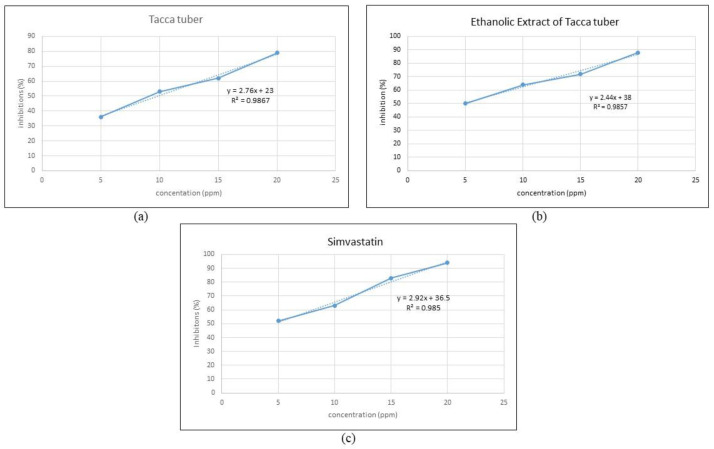
Inhibitory activity of HMG-CoA reductase of Tacca tuber (**a**), ethanolic extract of Tacca tuber (**b**) and simvastatin (**c**).

The IC_50_ value indicates the potential for inhibition of the HMG-CoA reductase enzyme, where the smaller the IC_50_ value, the greater the inhibition potential for the activity of the HMG-CoA reductase enzyme [48]. In vitro testing using the HMG-CoA reductase assay kit showed that the ethanol extract from Tacca tubers acted as an inhibitor of HMG-CoA reductase with an IC_50_ value of 4.92 ppm, which was close to the IC_50_ value of simvastatin by comparison (4.62 ppm). The results of this study indicate that the higher the concentration, the greater the inhibition of HMG-CoA reductase, as shown in Figure 5. Tacca tubers, the ethanol extract from Tacca tubers and simvastatin have inhibitory activities against HMG-CoA reductase of 79%, 88% and 94%, respectively, at a concentration of 20 ppm. The inhibitory activity of the HMG-CoA reductase enzyme from the ethanol extract of Tacca tubers was lower than simvastatin, therefore it is concluded that the ethanol extract from Tacca tubers has the potential to inhibit cholesterol synthesis, although not as well as simvastatin. Carbonell & Freire [49] stated that plant extracts capable of inhibiting the activity of the HMG-CoA reductase enzyme may function as hypocholesterolemic agents.

## 3. Materials and Methods

### 3.1. Materials

**Figure 6:**
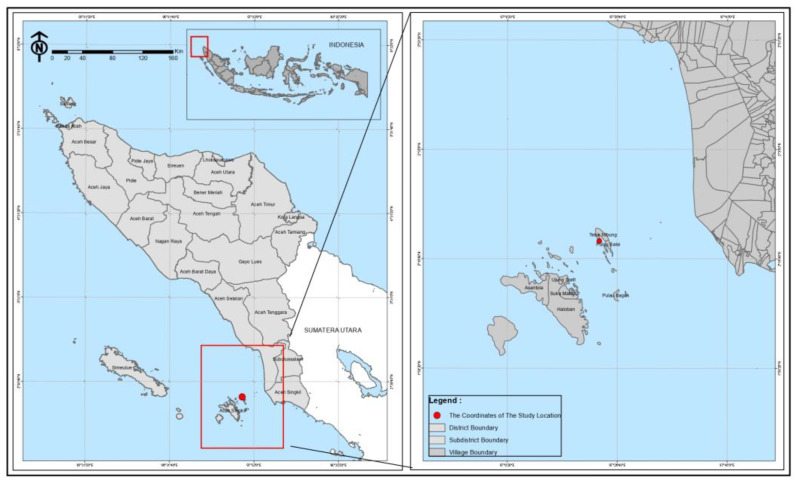
Sampling location map.

The plant materials, namely Tacca tubers (*T. leontopetaloides*), were collected from Teluk Nibung village, Ujung Batu Island, Banyak Islands, Aceh Singkil, Aceh Province. The sampling location was 2°20′54″ N 97°23′08″ E (Figure 6). The materials used in this study were distilled water, ethanol 96%, Dragendorff reagent, Wagner reagent, Meyer reagent, FeCl_3_ (Merck & Co, Rahway, NJ, USA), magnesium powder, HCl (Merck), chloroform (Merck), anhydrous acetic acid, H_2_SO_4_ (Merck), Folin–Ciocalteau reagent (Merck), Na_2_CO_3_ (Merck), NaNO_2_ (Merck), AlCl_3_ (Merck) and NaOH (Merck). The 3D structure of the HMG-CoA reductase enzyme (PDB ID: 2R4F) and the 3D structure of the ligand were downloaded from the Protein Data Bank (PDB).

This research utilized gas chromatography–mass spectrometry (GC-MS), a computer (hardware specifications: Intel Celeron 4205U processor Dual core 1.8 GHz base frequency, 4GB RAM, Windows 10 Home ×64 operating system) and the web-based software Autodock 4.0 on a docking server (http://www.dockingserver.com, accessed on 3 January 2022).

### 3.2. Methods

#### 3.2.1. Preparation of Tuber Extract

**Figure 7:**
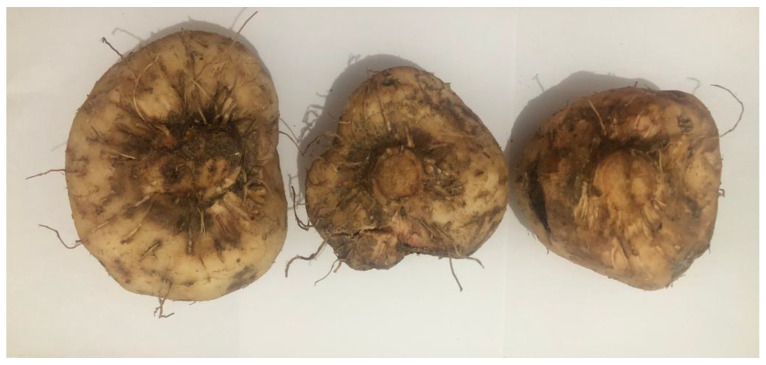
Tuber of *Tacca leontopetaloides*.

Samples of the Tacca plant (*T. leontopetaloides*) were determinations in the Department of Biology, Faculty of Mathematics and Natural Sciences, University of Syiah Kuala (USK) Banda Aceh. The samples in this study were Tacca tubers with weights of ±300 g (Figure 7). Samples of Tacca tubers were peeled and cut into pieces before the extraction process. Then the samples were dried using an oven at a temperature of 35–40 °C. The extraction process was carried out by the maceration method 3 × 24 h using ethanol 96%, with a ratio of 1:10. The samples in the form of mashed Tacca tubers (simplicia) were put into maceration bottles (macerator). Solvent was then added until all the ingredients were submerged and allowed to stand for 3 days in a place protected from sunlight while stirring occasionally. After 3 days, filtering was carried out using filter paper so that the macerated sample and pulp were obtained, which were then re-maceration twice. The resulting macerated sample was then concentrated using a Rotary Evaporator (Buchi Rotavapor^®^ R-300, Flawil, Switzerland) to produce a concentrated extract [50].

#### 3.2.2. Phytochemical Test

Qualitative testing of the phytochemical compounds from the ethanolic extract of Tacca tuber includes testing for alkaloids, flavonoids, steroids, terpenoids, phenolics, tannins and saponins, following the general instructions by Harbone [51]. The saponin test was carried out by checking the stale foam after shaking using distilled water. The steroid and terpenoid test was conducted using the Liberman-Burchard reagent, whereas for the alkaloid test, we used Mayer, Dragendorff and Wagner reagents. The phenolic test used FeCl_3_, the flavonoid test used Mg powder and the tannin test used gelatin and sulfuric acid [52].

#### 3.2.3. Identification of Active Compounds Using Gas Chromatography-Mass Spectrometry (GC-MS)

A total of 5 µL of ethanolic extract from a Tacca tuber was injected into the GC-MS instrument (Agilents Technologies 7890GC/5975MS) using an HP Ultra 2 capillary column with a length of 30 m, inner diameter of 0.20 mm and film thickness of 0.11 µm, and helium as the carrier gas with a constant column flow of 1.2 mL/min and split ratio of 8:1. The oven temperature was initially set to a temperature of 80 °C (held for 0 min), then increased at 3 °C/min to 150 °C (held for 1 min) and then finally increased at 20 °C/min to 280 °C (held for 26 min). The injection site temperature was 250 °C, the ion source temperature was 230 °C and the interface temperature was 280 °C. The eluted component was detected on the mass detector. The mass spectrum fragmentation pattern was compared with that stored in the spectrometer database, which uses the W8N08 Mass Spectral library. The percentage of each component was calculated from the relative peak area of each component in the chromatogram [53].

#### 3.2.4. Molecular Docking (In Silico)

Molecular docking was carried out in several stages, namely, protein preparation, ligand preparation, docking and analysis [54,55,56,57].

1. Preparation of target proteins (receptors)

The target protein used was HMG-CoA reductase. The 3D structure of the HMG-CoA reductase enzyme can be downloaded from the Protein Data Bank (PDB) at www.rcsb.org, accessed on 3 January 2022. The downloaded protein was removed the water molecule and optimized. We then determined the active site of the HMG-CoA reductase enzyme. The target protein was then prepared using BIOVIA Discovery Studio software 2021 and saved in the PDB format.

2. Preparation of ligands

The active compounds selected as test ligands to be attached to the target protein. The chosen control ligand is the simvastatin one of the HMG-CoA reductase enzyme. Ligands can be downloaded through the PubChem database, prepared with OPEN BABEL Sketch and then saved in PDB format.

3. Determination of the active site

The active side is where the ligand and compound will bind. Determination of the active side is done with Autodock 4.0.

4. Molecular docking

At this stage, the docking between the target protein (HMG-CoA) with the ligand and the comparison compound simvastatin is carried out. The docking process is carried out using AutoDock Vina software with PyRx emulator software. The ligand molecule will interact on the active site of the receptor and will then inhibit the function of the receptor. Finally, the ligand is able to act as a drug.

5. Analysis

The next stage is the analysis or interpretation of the results by looking at the type of molecular bond interactions that occur between the protein and the ligand.

#### 3.2.5. In Vitro HMG Co-A Reductase Inhibitory Activity [58,59]

HMG-CoA reductase inhibitory activity was analyzed using the HMG-CoA reductase (HMGCR) enzymatic assay kit (Sigma Aldrich, Catalog No. CS1090). The kit consists of an assay buffer, NADPH, HMG-CoA as the substrate solution, HMG-CoA reductase (HMGCR) as the catalytic domain (0.5–0.7 mg/mL) and pravastatin as an inhibitor solution. The inhibitory activity of the ethanolic extract on HMG-CoA reductase (HMGCR) activity is monitored by comparing it to pravastatin (kit inhibitor) and simvastatin (commercial inhibitor), which serve as references. The Tacca tuber, ethanolic extract and simvastatin were tested at four concentrations: 5, 10, 15 and 20 ppm. The assay reaction was prepared by mixing 1× assay buffer, pravastatin, NADPH, HMG-CoA and HMGCR, according to the procedure of the assay kit. The activity of the HMG-CoA enzyme was measured immediately with a Micro-plate/ELISA Reader UV/Vis spectrophotometer every 10 s for 10 min at 37 °C and λ = 340 nm. Enzyme activity is expressed in units/mg of protein and calculated by Equation (1).

(1) $$Unit/mgP=\frac{\left(\frac{A340}{min_{sample}}-\frac{A340}{min_{blank}}\right)\times TV}{12.44\times V\times 0.6\times LP}$$

where:

*A*340 = absorbance

*TV*  = total volume (mL)

*V*       = volume of enzyme used

*LP*     = light path (cm)

0.6  = enzyme concentration in mg protein/mL

12.44 = NADPH requirement during reaction (coefficient for NADPH at 340 nm is 6.22/mM cm)

The percentage of inhibition of HMGCR was calculated using the equation:

(2) $$\mathrm{Inhibitor}\;\left(\%\right)=\frac{\left(enzyme\;activity\;-\;sample\;activity\right)}{enzyme\;activity}\;\times \;\;100\%.$$

The IC_50_ value is the inhibitor concentration required to achieve 50% HMG-CoA reductase inhibition. Determination of the IC_50_ value was completed using linear regression to get the slope value of the variable, or non-linear regression using the sigmoidal equation, where the independent variable is the concentration of the inhibitor and the dependent variable is the percentage of inhibition.

## 4. Conclusions

The HMG-CoA reductase activity of the ethanolic extract of Tacca tuber was inhibited by in vitro, with an IC50 value of 4.92 ppm. The stigmasterol (−7.2 Kcal/mol) component of the phytosterols is the substance from GCMS of the ethanolic extract of the tacca tuber that shows inhibitory activity against HMG-CoA reductase (PDB ID: 2R4F). Simvastatin is used as a positive control, and it has a binding affinity of −8.0 Kcal/mol and an IC50 value of 4.62 ppm. Based on the results, it can be concluded that the ethanolic extract from Tacca tubers has the potential to inhibit cholesterol by inhibiting the activity of HMG-CoA reductase.

## Figures and Tables

**Table 1:** Taxonomic Classification of Tacca Plant.

Taxonomic Rank	Taxon
Regnum/kingdom Sub regnum/sub kingdom:	*Plantae* *Tracheobionta*
Super divisio/super division:	*Spermatophyta*
Divisio/division:	*Magnoliophyta*
Classis/class:	*Liliopsida*
Sub classis/sub class:	*Liliidae*
Ordo/order:	*Liliales*
Familia/family:	*Taccaceae*
Genus/genus:	Tacca J.R. Forst. & G. Forst
Species/species:	*Tacca leontopetaloides* (L.) Kuntze

**Table 2:** The active compounds in ethanolic extract from Tacca tuber (*T.leontopetaloides*) by GC-MS.

No	Retention Time (RT)	Area (%)	Name of the Compound	Quality (%)
1	7.12	1.16	2,3-Dihydro-3,5-dihydroxy-6-methyl-4H-pyran-4-one	94
2	10.24	2.12	Furancarboxaldehyde, 5-hydroxymethyl	90
3	26.82	1.23	1-Octadecene	98
4	27.92	3.33	1-isopropyl-2-methoxycarbonyl-1-aza-cyclopropane	18
5	28.56	1.02	Silane, ethoxytrimethyl	46
6	28.76	3.98	Hexadecanoic acid	99
7	29.39	1.75	Methyl(9Z,12Z)-9,12-octadecadienoate	99
8	29.58	0.77	E-8-methyl-9-tetradecen-1-ol-acetate	91
9	29.86	7.00	9,12-octadecadienoic acid	99
10	30.70	1.34	Beta-pinone	46
11	30.91	2.12	Hexanedioic acid, BIS(2-ethyhexyl) ester	81
12	31.21	1.03	ZZ, 10,12-hexadecadien-1-ol-acetat	55
13	31.43	0.97	6,8-Dioxabicyclo(3,2,1)OCT-3-ENE	64
14	31.78	44.39	1,2-Benzenedicarboxylic acid, dinonyl ester	91
15	32.15	0.85	1,9,12,15-octadecatetraene-1-methoxy	51
16	32.93	2.74	Phthalic acid, bis (7-methyloctyl) ester	80
17	33.31	10.91	1,2 Benzenedicarxylic acid, mono (2-ethylhexyl) ester	50
18	37.58	0.62	Campesterol	99
19	38.06	0.78	Stigmasterol	99
20	39.06	1.53	Gamma sitosterol	99

**Table 3:** Molecular docking of the active compounds in the ethanolic extract from Tacca tuber and simvastatin.

Ligan (The Active Compounds)	Binding Affinity (Kcal/mol)	Amino Acid Residue
Simvastatin	−8.0	Van der Waals: ASP516, PRO513, ARG515, GLN510, TYR514, PRO813 Hydrogen: ARG515, ASP516, TYR533 Alkyl: PRO535, TYR517, PRO513, TYR511
2,3-Dihydro-3,5-dihydroxy-6-methyl-4H-pyran-4-one	−5.3	Van der Waals: ASN518, TYR533, LEU812, TYR511 Hydrogen: ASP516 Alkyl: PRO535, TYR533, PRO813, TYR511
Furancarboxaldehyde, 5-hydromethyl	−5.5	Van der Waals: ALA585, THR636, LYS606, LEU584, ILE638, PRO798, ILE699, TRP698, GLY701 Hydrogen: SER705, GLU700 Carbon hidrogen: SER637
1-octadecene	−4.5	Van der Waals: THR809, GLN766, GLY765, ASP767, GLY807, GLU559 Hydrogen: GLY808, THR558 Alkyl: MET655
1-isopropyl-2-methoxycarbonyl-1-aza-cyclopropane	−5.2	Van der Waals: TYR514, TYR533, ARG515, ASP516, TYR517, PRO535, PRO813, TYR511, GLN510, LEU812, ASN518 Alkyl:PRO513
Silane, ethoxytrimethyl	−7.6	Van der Waals: ARG496, TYR514, ARG515, ASP516, TYR533, TYR517, PRO535, PRO813, TYR511, GLN510, ASN518 Alkyl: PRP513
Hexadecanoic acid	−4.4	Van der Waals: ASN518, TYR517, ARG51, PRO813, PRO513, PRO535 Hydrogen: TYR533, ASP516
Methyl(9Z,12Z)-9,12-octadecadienoate	−4.0	Van der Waals ILE733, GLU730, GLU726, GLU789, ASN788, THR725 Hydrogen: ASN788 Alkyl: ILE729, ALA783
E-8-Methyl-9tetradecen-1-ol-acetate	−6.5	Van der Waals:GLY491, ILE485,ILE494, ILE467, LYS474, LEU452, ASN445 Unfavourable donor: ARG495 Alkyl: VAL471, LEU470, ALA473, LEU449
9,12-octadecadienoic acid	−5.0	Van der Waals: PRO813, PRO535, PRO513, TYR533, TYR511, ASP516, ASN518, TYR517
Beta pinone	−6.4	Van der Waals: CYS568, GLU528, ASN529, ASN567, LYS474, PRO477, ALA478 Pi-Sigma: TYR479 Alkyl: AL564
ZZ,10,12-hexadecadien-1-ol-acetate	−4.0	Van der Waals: GLU726, GLU730, ASN788, ILE7333, GLU782, ASN734 Hydrogen: MET781 Alkyl: ILE729, ALA783
6,8-Dioxabicyclo(3,2,1)OCT-3-ENE	−4.8	Van der Waals: ASP516, ARG515, TYR533, TYR517, TYR511, ASN518, LEU812, PRO535 Alkyl: ARG515, TYR514, TYR533, PRO513, PRO813
1,2 Benzenedicarboxylic acid, dinonil ester	−4.9	Van der Waals: MET781, ASN734, GLU782, GLU730, ARG595, ASN788, Hydrogen: GLU730 Alkyl: LEU780, ILE733, LEU737, ILE729, ALA783
1,9,12,15, octadecatetraene-1-methoxy	−4.3	Van der Waals: ALA585, ILE699, GLU700, THR636, SER637, MET742, THR796, PRO798, LEU584, SER705, LYS606, GLY701 Hydrogen: ILE638
Campesterol	−4.9	Van der Waals: ASN755, GLY560, CYS561, GLU559, LYS735, ARG590, LYS691, ALA751, ASP690 Hydrogen: SER684, LYS692 Alkyl: LEU853, HIS752, LEU562, LEU857, VAL683, ALA856
Stigmasterol	−7.2	Van der Waals: ASN518, ASP516, PRO535, ARG515, TYR533, TYR514, GLN510 Alkyl: TYR533, PRO813, TYR511, TYR517, PRO513, ARG515
Gamma sitosterol	−4.5	Van der Waals: LYS633, HIS63, ARG646, GLN632, GLN648, LYS606, THR636, GLY701, SER705, SER637, MET742, PRO798, THR796 Unfavorable donor: ILE638 Alkyl: ALA585, LEU584

## Data Availability

The data in this study are available upon request to the author.

## Sample Availability

Samples of the compounds are available from the authors.
